# Construction of Metal–Organic Framework as a Novel Platform for Ratiometric Determination of Cyanide

**DOI:** 10.3390/bios14060276

**Published:** 2024-05-27

**Authors:** Zongbao Sun, Zhiwei Wu, Yiran Zong, Chen Li, Wang Guo, Yiqing Guo, Xiaobo Zou

**Affiliations:** School of Food and Biological Engineering, Jiangsu University, Zhenjiang 212013, China; 17354287873@163.com (Z.W.); zongyiran1999@163.com (Y.Z.); 13529128159@163.com (C.L.); wguo6786@gmail.com (W.G.); 18632927732@163.com (Y.G.); zou_xiaobo@ujs.edu.cn (X.Z.)

**Keywords:** MOF, electrochemical sensor, cyanide, TCPP, Fc−COOH

## Abstract

Metal–organic frameworks (MOFs) are frequently utilized as sensing materials. Unfortunately, the low conductivity of MOFs hinder their further application in electrochemical determination. To overcome this limitation, a novel modification strategy for MOFs was proposed, establishing an electrochemical determination method for cyanides in Baijiu. Co and Ni were synergistically used as the metal active centers, with meso−Tetra(4−carboxyphenyl)porphine (TCPP) and Ferrocenecarboxylic acid (Fc−COOH) serving as the main ligands, synthesizing Ni/Co−MOF−TCPP−Fc through a hydrothermal method. The prepared MOF exhibited improved conductivity and stable ratio signals, enabling rapid and sensitive determination of cyanides. The screen−printed carbon electrodes (SPCE) were suitable for in situ and real−time determination of cyanide by electrochemical sensors due to their portability, low cost, and ease of mass production. A logarithmic linear response in the range of 0.196~44 ng/mL was demonstrated by this method, and the limit of detection (LOD) was 0.052 ng/mL. Compared with other methods, the sensor was constructed by a one−step synthesis method, which greatly simplifies the analysis process, and the determination time required was only 4 min. During natural cyanide determinations, recommended readouts match well with GC−MS with less than 5.9% relative error. Moreover, this electrochemical sensor presented a promising method for assessing the safety of cyanides in Baijiu.

## 1. Introduction

Cyanide is a toxic compound containing a cyanide group (−CN) or cyanide ion (CN^−^), such as KCN, NaCN, and HCN [1]. Cyanide is highly toxic, causing cell suffocation and death with blood flow [2]. Specific conditions, such as the presence of cyanogenic glycosides [3,4] in raw materials or the utilization of water sources and auxiliary materials contaminated with cyanide compounds, can lead to an increase in the cyanide content in alcoholic beverages. Baijiu is a type of alcoholic beverage that is very popular in China. During the high−temperature fermentation of Baijiu, cyanides are hydrolyzed and then react with ethanol, resulting in the formation of another carcinogen, ethyl carbamate [3,4]. According to national food safety standards, the cyanide content is limited to a maximum of 8 mg/L (calculated as HCN). In the updated “Detailed Rules for the Examination of Baijiu Production Licenses”, it is emphasized that the capability to conduct self−inspections of key food safety indicators, including cyanides and ethyl carbamate, should be possessed by enterprises. The monitoring of cyanide concentrations is instrumental in the assessment and control of potential health risks in alcoholic products.

In laboratories, the determination of cyanides in food is primarily conducted using ion chromatography [5], gas chromatography−mass spectrometry [6], and liquid chromatography [7]. Electrochemical sensors are widely used due to their advantages such as fast response, simple operation, high sensitivity, and low cost [8]. These sensors exhibit adequate precision and accuracy, and their instruments are relatively portable. Owing to their cost−effectiveness and potential for miniaturization [9], electrochemical sensors are considered a promising point−of−care (POC) platform. The stability and repeatability of the sensors can be greatly ensured by the proportional strategy [10]. Ratiometric electrochemical sensors quantify the analyte with a record ratiometric of two signals (one is from the analyte and the other is from the inner reference). A peak intensity ratio (*I*_analyte_/*I*_inner_ reference) is used as the measurement criteria for analytes [11].

Metal–Organic Frameworks (MOFs) are recognized as periodic network−structured organic–inorganic hybrid materials, assembled through coordination bonds between organic ligands and metal ions/clusters [12]. MOFs show high specific surface areas, rich porosity, excellent redox activity, and customizable structure and functionality [13,14,15,16,17]. The problem of poor electrical conductivity can be improved by metal doping combined with ligand regulation, which helps to adjust the composition elements, morphology, specific surface area, and pore size of MOFs [18], as well as improve their catalytic and electrochemical properties [19]. For example, ultrathin Co/Ni− MOF nanosheets were used as a platform for luminol−functionalized AgNPs to construct an immunosensor for tumor marker alpha−fetoprotein [20]. Bimetallic MOFs with superior electrochemical performance grown on electrospun nanofibers (PPNF@M−Ni MOF, M = Co, Zn, Cu, Fe) were prepared by controlling the incorporation of various types of metal ions [21]. ZnZr bimetallic MOFs were prepared as an aptasensor platform for detecting the cancer marker protein tyrosine kinase−7 (PTK7) [22]. The appropriate selection of metal ions and ligands is of fundamental importance to improve the electrochemical performance of MOFs. Ferrocene (Fc) and its derivatives are known for their unique redox and electrocatalytic properties [23,24,25], while porphyrins are known for their large π−conjugated structures and excellent photoelectric properties [26,27,28,29]. Ferrocene (Fc) and its derivatives and porphyrins all show excellent ligands for MOF.

**Figure 1:**
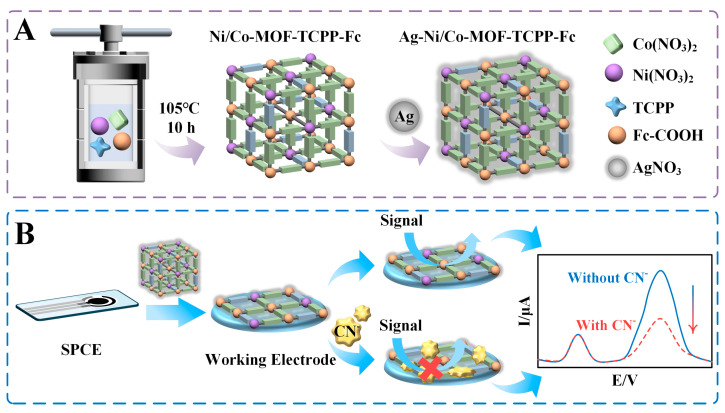
(**A**) Preparation procedure of Ni/Co−MOF−TCPP−Fc. (**B**) Schematic representation of electrochemical sensing of CN^−^ by using Ni/Co−MOF−TCPP−Fc/SPCE.

In the present work, ferrocene carboxylic acid (Fc−COOH) and porphyrin (using TCPP as the doping ligand) are introduced into Ni/Co−MOF to enhance the electrochemical performance of Ni/Co−MOF (Figure 1A). Fc−COOH provides an additional metal catalytic center (Fe) that promotes the production of reactive oxygen species. Together with porphyrin as ligands, a greater number of unsaturated sites are formed, enhancing the electro−conductivity of Ni/Co−MOF−TCPP−Fc. After synthesis of Ag on the surface, the metals in Ag−Ni/Co−MOF−TCPP−Fc react with cyanide to form a metal complex, leading to inhibition of the electron transfer on the electrode surface. As the concentration of cyanides increases, the complexation reaction is intensified, leading to a decrease in the electrochemical signal, while the reference signal remains stable (Figure 1B). The ratio value of *I*_CN−_/*I* serves as an excellent indicator for cyanide evaluation. This sensor greatly simplifies the analysis process, improves the determination efficiency, and exhibits the potential to be used for cyanide monitoring in Baijiu.

## 2. Materials and Methods

### 2.1. Main Reagents and Materials

Potassium chloride (KCl), anhydrous ethanol, potassium ferricyanide (K_3_[Fe(CN)_6_]), potassium ferrocyanide (K_4_[Fe(CN)_6_]), and other reagents were supplied by Sinopharm Chemical Reagent Co., Ltd. (Shanghai, China). Polyvinylpyrrolidone, terephthalic acid, ferrocene carboxylic acid (Fc−COOH), N,N−Dimethylformamide (DMF), 2−methylimidazole (2−Mim), Cobaltous nitrate hexahydrate (Co(NO_3_)_2_·6H_2_O), and nickel nitrate hexahydrate (Ni(NO_3_)_2_·6H_2_O) were provided by Shanghai Aladdin Biochemical Technology Co., Ltd. (Shanghai, China). Meso−tetra(4−carboxyphenyl)porphine (97%) was supplied by Shanghai Macklin Biochemical Technology Co., Ltd. (Shanghai, China). Phosphate buffered saline (PBS, 0.1 M, pH 7.4) and cyanide standards in water were provided by Shanghai Anpel Laboratory Technologies Co., Ltd. (Shanghai, China). The water used was ultrapure water. Screen−Printed Carbon (SPCE) Electrode was purchased from Poten (Beijing, China).

### 2.2. Main Instruments and Equipment

Composite microstructures were explored with scanning electron microscopy (SEM, JSM−7800M, JEOL, Tokyo, Japan). Bonding characteristics were examined with an X−ray photoelectron spectrometer (XPS, K−Alpha, Thermo, Waltham, MA, USA), using Al−Kα radiation with 1486.6 eV energy. An X−ray diffractometer (XRD, D8 ADVANCE, Bruker, Bremen, Germany) was employed with crystal phases of TCPP, Fc−COOH, and Ni/Co−MOF−TCPP−Fc at a 2θ angle from 5° to 80° with a Cu−Kα radiation source (λ = 1.5405 Å). Fourier transform infrared spectra (FT−IR) were studied with an FT−IR analyzer (Nicolet is50, Nicolet, Wausau, WI, USA). N_2_ physisorption isotherms were measured on a surface area analyzer (NOVA3000e, Quantachrome, Boynton Beach, FL, USA), in which the Brunauer–Emmett–Teller (BET) model was applied to estimate specific surface area and the Barrett–Joyner–Halenda (BJH) model was employed to analyze pore size. All electrochemical measurements including differential pulse voltammetry (DPV) and cyclic voltammetry (CV) were evaluated with a CHI potentiostat (660E, Shanghai Chen Hua Instrument Co., Ltd., Shanghai, China).

### 2.3. Preparation of Electrochemical Sensor

Ni/Co−MOF−TCPP−Fc was synthesized via a hydrothermal method following the previously reported procedures with slight modifications [30,31]. A total of 0.1866 g Co(NO_3_)_2_·6H_2_O and 0.2958 g Ni(NO_3_)_2_·6H_2_O, along with 0.5 g polyvinylpyrrolidone, were dissolved in 60 mL solution including DMF and ethanol mixture (3:1, *v*/*v*) to prepare solution A. Subsequently, 200 mg TCPP was dissolved in 20 mL solution including DMF and ethanol mixture (3:1, *v*/*v*) with thorough stirring until fully dissolved, resulting in solution B. Then, 0.6646 g terephthalic acid and 0.05 g Fc−COOH were dissolved in 20 mL DMF, and the mixture was stirred for 25 min until fully dissolved to obtain solution C.

Then, under vigorous stirring, solutions B and C were added dropwise to solution A. After ultrasonication of the mixture for 25 min, it was transferred to a 100 mL polytetrafluoroethylene−lined autoclave and reacted at 105 °C for 10 h. Upon naturally cooling to room temperature, the solid was collected by centrifugation (4000 rpm, 10 min), washed several times alternately with DMF and anhydrous ethanol, and then dried at 60 °C for 12 h to obtain the Ni/Co−MOF−TCPP−Fc. Then, 100 mg Ni/Co−MOF−TCPP−Fc was dispersed in 50 mL of distilled water and mixed ultrasonically with AgNO_3_ (10 mL 2 mM) solution, and then NaBH_4_ solution (5 mL 10 mM) was added drop by drop during agitation. Ag−Ni/Co−MOF−TCPP−Fc was obtained after being magnetically stirred at room temperature for 2 h, washed with ethanol three times, and dried at 60 °C. A total of 10 µL of the prepared Ag−Ni/Co−MOF−TCPP−Fc (1 mg/mL) was drop−coated onto the surface of a screen−printed carbon electrode (SPCE) and dried at room temperature to obtain the Ag−Ni/Co−MOF−TCPP−Fc/SPCE.

### 2.4. Electrochemical Experiment

A three−electrode system was used: a working electrode (material: carbon, electrode diameter: 4 mm), a counter electrode (material: carbon), and a reference electrode (material: Ag/AgCl). Gradual modification of the electrode was verified by CV and DPV. The CV scan rate was set to 100 mV/s, with scanning windows of −0.6~1 V. In DPV measurements, the potential window, pulse amplitude, pulse width, and pulse period were set to −0.8~0.6 V, 50 mV, 16.7 ms, and 100 ms, respectively.

### 2.5. Determination of Real Samples

Natural samples including Baijiu, fermented grains, river water, and mineral water were involved to examine the proposed method. Three kinds of Baijiu (sauce−flavor, strong−flavor, and light−flavor) and mineral water were purchased at Jimailong supermarket (Zhenjiang, China). Baijiu samples were diluted to less than 20% alcohol to be measured. Mineral water samples could be directly used for detection. River samples were collected from the campus. They were centrifuged (10,000 rpm, 10 min) and filtered with 0.22 µm microporous membrane to remove undissolved substances. Fermented grains were provided by a winery in Jiangsu Province. In a centrifuge tube, 1 g fermented grains and 50 mL pure water were sonicated for 20 min and then centrifuged for 5 min (4000 rpm). The supernatant was taken for testing. All samples were stored at 4 °C before further analysis. These samples were tested by dropping them onto the Ag−Ni/Co−MOF−TCPP−Fc/SPE sensor.

These samples were also examined with Gas Chromatography−Mass Spectrometry (GC−MS, TQ8040, SHIMADZU, Kyoto, Japan) and recovery assays concurrently. Prior to GC−MS analyses, pretreated samples were filtered with a 0.22 µm microporous membrane. The analytical column was a capillary column (30 m × 0.25 mm id × 0.25 μm film thickness). The flow rate of helium as carrier gas was 1.0 mL/min. The oven temperature program began at 40 °C (held for 2 min), was raised to 60 °C (held for 2 min) at 20 °C/min, and then increased to the final temperature of 200 °C at 50 °C/min (held for 2 min). The inlet temperature was 200 °C, the shunt ratio was 10:1, and the column flow rate was 1.5 mL/min. The ion source temperature of the mass spectrometry was 230 °C, the interface temperature was 220 °C, and the solvent delay time was 1 min. The monitoring mode was ion scanning, the scanning ion *m*/*z* was set to 61, 63, and 35, and the quantitative ion *m*/*z* was set to 61.

During the recovery test, a pretreated natural sample was analyzed with the proposed sensor to obtain the original CN^–^ amount (the 1st analysis). Then, it was spiked with 0.1 µg/mL CN^−^ for the 2nd analysis.

## 3. Results and Discussion

### 3.1. Morphological Study and Chemical Composition of Ni/Co−MOF−TCPP−Fc

**Figure 2:**
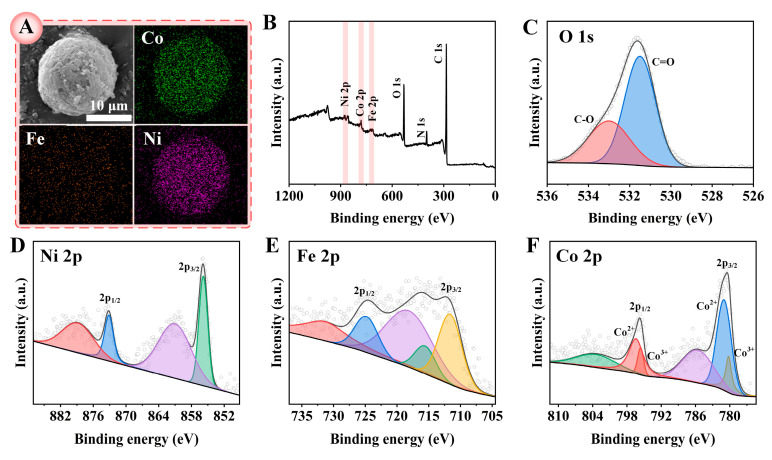
(**A**) SEM of Ni/Co−MOF−TCPP−Fc and elemental mappings of Co, Fe, and Ni. (**B**) XPS spectrum of Ni/Co−MOF−TCPP−Fc. The high−resolution XPS spectra of (**C**) O 1s, (**D**) Ni 2p, (**E**) Co 2p, and (**F**) Fe 2p.

The Ni/Co−MOF−TCPP−Fc, which was synthesized via the direct hydrothermal method, exhibited an irregular block−like structure with small particles on its surface, leading to a relatively rough texture. The size of Ni/Co−MOF−TCPP−Fc was in the range of 14~25 µm. This texture is beneficial for increasing catalytic sites. As evidenced by the SEM image, small particles were prominently present on the surface of the material. These small particles were revealed to be compounds of Co and Ni through surface characterization using XPS and mapping. A uniform distribution of Fe, Ni, and Co atoms within the Ni/Co−MOF−TCPP−Fc was demonstrated by elemental mapping (Figure 2A).

The elemental composition of Ni/Co−MOF−TCPP−Fc was further analyzed with an X−ray photoelectron spectrometer. The presence of Fe, Co, Ni, O, H, and C elements in the full XPS spectrum was consistent with the results of the mapping analysis (Figure 2B). The O 1s spectrum of the high−resolution XPS, shown in Figure 2C, corresponded to C=O and C−O for binding energies at 531.49 eV and 532.99 eV, respectively, which were mainly derived from the carboxyl group of the terphenyl acid ligand [32]. The presence of Ni^2+^ was confirmed by the Ni 2p_3/2_ (855.79 eV) and Ni 2p_1/2_ (873.09 eV) peaks in the Ni 2p high−resolution XPS map (Figure 2D), and the peaks with binding energies of 879.09 eV and 861.29 eV were satellite peaks of Ni [33]. The binding energies at 711.69 eV and 725.09 eV correspond to Fe 2p_3/2_ and Fe 2p_1/2_, respectively, both from Fe^2+^, Fe^3+^ characteristic peaks in the ferrocene structure, and the introduction of Fc−COOH was confirmed (Figure 2E). The Co 2p spectrum of high−resolution XPS is shown in Figure 2F. The Co 2p emission spectrum had two spin−orbit duplex states, with Co^2+^ ions with binding energies of 781.19 eV and 796.59 eV and Co^3+^ ions with binding energies of 780.19 eV and 795.59 eV. There were two oscillatory satellite peaks of 803.79 eV and 785.79 eV, respectively [34].

### 3.2. Synthetic Properties of Ni/Co−MOF−TCPP−Fc

**Figure 3:**
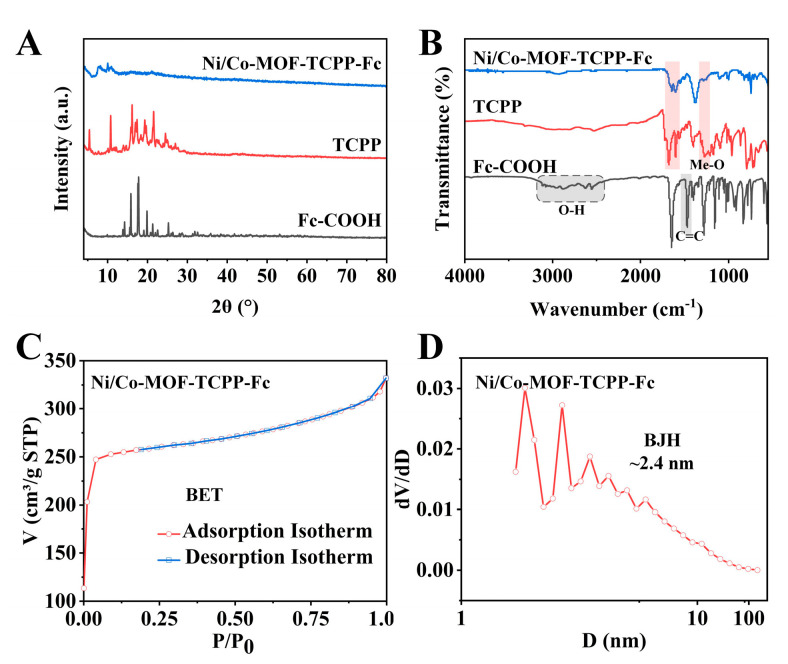
(**A**) XRD patterns and (**B**) FT−IR spectra of Ni/Co−MOF−TCPP−Fc, TCPP, and Fc−COOH. (**C**) Nitrogen adsorption–desorption isotherms and (**D**) the corresponding pore size distribution of Ni/Co−MOF−TCPP−Fc.

The crystal structure of Ni/Co−MOF−TCPP−Fc was characterized using XRD, and as shown in Figure 3A, the results were similar to those previously reported for Ni/Co−MOF [34]. The arrangement of the metals Co and Ni as framework nodes within the crystal structure is indicated by the absence of distinct metal peaks in Ni/Co−MOF−TCPP−Fc. Additionally, the crystallinity of Ni/Co−MOF may be affected by the incorporation of the ligands Fc−COOH and TCPP, leading to a deterioration in the crystal form and thus exposing more active sites.

The coordination between the ligands and the MOF material was analyzed using FT−IR (Figure 3B). The attribution of the absorption peak at 1006 cm^−1^ in Ni/Co−MOF−TCPP−Fc to the stretching vibration of the Me−N bond was indicative of the coordination between metal atoms and N atoms, suggesting that the formation of Co−N [35] and Ni−N [36] bonds was likely to enhance the enzyme−like catalytic activity of the material. The formation of metal–carboxylate bonds was proven by attributing the absorption bands between 1630~1480 cm^−1^ to the asymmetric and symmetric stretching vibrations of the carboxylate group (−COO−) that was bound to metals (Co or Ni) after coordination. For Ni/Co−MOF−TCPP−Fc, the absorption peak of TCPP at 1270 cm^−1^ was weakened, indicating that the hydrogen on −OH was replaced by metal ions and formed an Me−O bond. The reduction in peak intensity at 1600 and 1690 cm^−1^ was possibly attributed to alterations in the electronic structure that occurred when porphyrin was incorporated as a ligand within the MOF [37,38]. A broad absorption band was observed for the pure substance of Fc−COOH between 3310~2500 cm^−1^ due to the O−H stretching vibration of the carboxyl group in Fc−COOH, and a stretching vibration peak of the C=C in cyclopentadiene was observed at 1475 cm^−1^ [39,40]. However, in Ni/Co−MOF−TCPP−Fc, a significant weakening of the absorption band and the disappearance of characteristic peaks were observed, indicating that coordination had been established between Fc−COOH and the metal ions (Co and Ni), instead of Fc−COOH entering the pores of MOFs in its free state.

Subsequently, the pore size and specific surface area of Ni/Co−MOF doped with Fc−COOH and TCPP were studied combined with nitrogen adsorption–desorption isotherms. The results are shown in Figure 3C. According to international Union of Pure and Applied Chemistry (IUPAC) classification, it belonged to type I adsorption isotherm, and there were many microporous structures in the material. The BET surface area of Ni/Co−MOF−TCPP−Fc was measured at 1111.35 m^2^/g, which endowed the material with enhanced catalytic performance due to its large specific surface area. The pore size distribution is shown in Figure 3C. The pore size distribution was not uniform, with a large number of 2.4 nm pores. The micropores of the material were analyzed, and a large number of 0.96 nm pores were included. The addition of Fc−COOH and TCPP to Ni/Co−MOF−TCPP−Fc resulted in a high surface area and abundant microporosity [41], enhancing the mass transfer efficiency in the catalytic process.

### 3.3. Electrochemical Characterization of Gradual Modification

**Figure 4:**
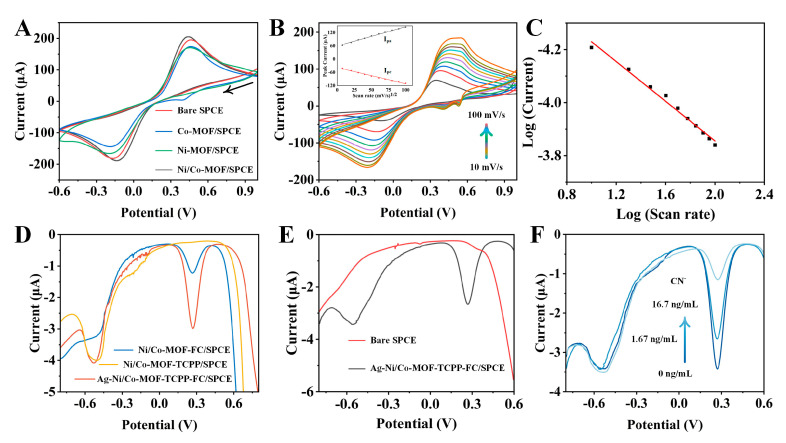
(**A**) CV curves (at 100 mV/s) of Co−MOF/SPCE, Ni−MOF/SPCE, and Ni/Co−MOF/SPCE in 5 mM K_3_[Fe(CN)_6_] and 0.1 M KCl. (**B**) Linear relationship between CV peaks and square roots of scan rates (10~100 mV/s, step: 10 mV/s) for Ag−Ni/Co−MOF−TCPP−Fc/SPCE in 5 mM K_3_[Fe(CN)_6_] and 0.1 M KCl. (**C**) Logarithmic fitting relationship curve for peak potential and scan rate. (**D**) DPV signals of Ni/Co−MOF−Fc/SPCE, Ni/Co−MOF−TCPP/SPCE, and Ag−Ni/Co−MOF−TCPP−Fc/SPCE in PBS buffer. (**E**) Signal response of bare electrode and modified electrode in PBS buffer (containing 1.67 ng/mL CN^−^). (**F**) Semiquantitative analyses in PBS buffer (containing 0, 1.67, and 16.7 ng/mL CN^−^).

The electrochemical performance of three types of MOFs (Co−MOF, Ni−MOF, and Ni/Co−MOF) was tested using a standard three−electrode system at a scan rate of 100 mV/s in a solution of 5 mM K_3_[Fe(CN)_6_] and 0.1 M KCl. From Figure 4A, within the −0.6~1 V range, the oxidation−reduction potentials of Co−MOF and Ni−MOF were relatively close. Due to the poor conductivity of MOF materials, the peak currents were weaker compared to the bare electrode. Under the cooperative effect of Co and Ni, in the presence of the ligands TCPP and Fc, the best CV response was exhibited by Ni/Co−MOF, with two reduction peaks appearing at −0.2 V and 0.46 V, indicating an enhancement in electrochemical activity. A more conductive electronic structure was formed as Fc−COOH acted as a defect−linker that can coordinate with metal ions through its carboxyl groups. Furthermore, the electron transport capability of MOFs was also improved by the introduction of the π−conjugated system by the TCPP ligand, enhancing the speed and current response of electrochemical reactions. Under scan rates ranging from 10 to 100 mV/s, it was shown that the oxidation−reduction signals of Ag−Ni/Co−MOF−TCPP−Fc significantly increased (Figure 4B). The peak currents of oxidation and reduction showed a good linear relationship with the scan rate, indicating a diffusion−controlled process on the electrode surface. Moreover, the peak separations begin to increase with increasing scan rate. This is consistent with the limitations which arise from charge transfer kinetics. This further confirmed that ion diffusion controls the electrochemical kinetics in the redox reaction (Figure 4C).

The DPV responses of electrodes modified with different materials were studied in 0.1 M PBS buffer. In Figure 4D, a distinct reduction peak at −0.5 V was observed for Ni/Co−MOF−TCPP, attributed to its polycyclic structure and multiple oxidizable redox centers, resulting in a significant current response. Two distinct reduction peaks at 0.26 V and −0.53 V (red curve) were produced by Ag−Ni/Co−MOF−TCPP−Fc, with the peak current reaching a maximum. This enhancement in the electrochemical response was due to the provision of abundant active sites by macrocyclic ligands such as TCPP and Fc to Ag−Ni/Co−MOF. Besides, for the blank solution, two significant peaks were displayed by the sensor. The bare electrode had no obvious electrical response to cyanide (Figure 4E). With the increase in cyanide concentration, the MOF peak was significantly suppressed, while the TCPP peak remained stable (Figure 4F). This phenomenon occurred because strong complexes with metal ions were formed by cyanide, which inhibited electron transfer on the electrode surface, leading to a reduction in the electrical signal.

Ag was the main chemical reaction element, and the metals Co and Ni were the secondary reaction ligands. The silver element may dissolve in the form of AgO and AgO^+^ in the anode scan. At the end of each CV cycle, it was reduced at the cathode in other forms of silver oxide and redeposited on the surface as Ag metal. The metal center of the MOF also participated in the reaction.

$$AgO+2{CN}^{-}+H_{2}O+e^{-}\to {\left[Ag{\left(CN\right)}_{2}\right]}^{-}+2{OH}^{-}$$

$$2Ag+4{CN}^{-}+\frac{1}{2}O_{2}+H_{2}O\to 2{\left[Ag{\left(CN\right)}_{2}\right]}^{-}+2{OH}^{-}$$

$${Co}^{2+}+6{CN}^{-}\to {\left[{Co(CN)}_{6}\right]}^{4-}$$

$${Ni}^{2+}+4{CN}^{-}\to {{Ni(CN)}_{4}}^{2-}$$

Hence, a proportional relationship was established between *I*_CN−_ and *I*.

### 3.4. Electrochemical Determination of CN^−^

The relationship between the current ratio and pH was showed in Figure 4. The current ratio gradually increased in the range of 2.8–7.4. This was because HCN was the main form in acidic environment, and acidic conditions inhibited the conversion of HCN to CN^−^. As pH increased, more HCN dissociated to form CN^−^. CN^−^ showed strong coordination ability and could form stable cyanogen complexes with various metal ions. Thus, in aiming to obtain better sensitivity for the assays of CN^−^, pH 7.4 was selected for the subsequent analysis.

**Figure 5:**
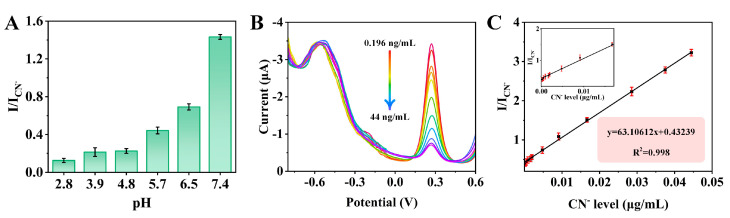
(**A**) pH optimization of PBS buffers (containing 16.7 ng/mL CN^−^). (**B**) DPV curves and (**C**) Linear calibration plots for laboratorial samples containing gradual−level of CN^−^ in 0.1 M PBS. Enlarged images in the 0~0.01 µg/mL is presented in the inset of (**C**).

Under optimal conditions, the DPV curves of the sensor were measured (Figure 5B). With the increase in cyanide concentration, a gradual decrease in the designed sensor *I*_CN−_ was observed, whereas *I* was found to remain almost unchanged. This phenomenon was attributed to the formation of stable metal complexes by metal ions with cyanide, which lack conductivity and hinder electron transfer on the electrode surface, further leading to a decrease in signal. In Figure 5C, a good linear relationship between *I*_CN_^−^/*I* and cyanide concentration in the range of 0.196~44 ng/mL was demonstrated by the standard curve, with a correlation coefficient of 0.998. The linear regression equation established was y = 63.10612x + 0.43239 (x is cyanide concentration), and the limit of detection (LOD) was 0.052 ng/mL (S/N = 3). Compared to previously reported sensors (Table 1), a better linear range and determination limit for cyanide were exhibited by the developed sensor.

### 3.5. Specificity and Stability of the Sensor

**Figure 6:**
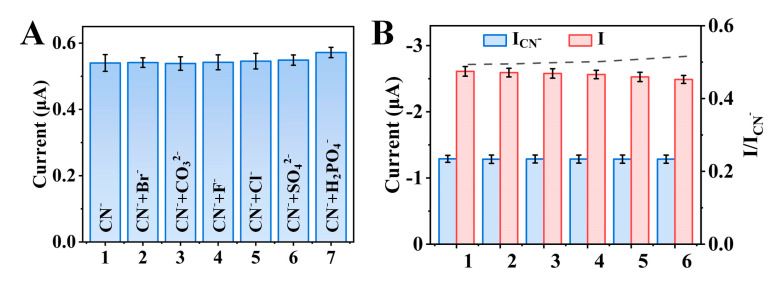
(**A**) DPV response at the sensor toward 0.1 ng/mL CN^−^ with the presence of different interference at 5 ng/mL. (**B**) Repeatability for the sensor. Recommended ratiometric readouts show better consistency than raw signals of *I*_CN_^−^ in experiments of (**B**) stability. All DPV readouts are conducted with 3 replicates.

Various ions existed in the Baijiu matrix. The selectivity of the sensor towards cyanide was investigated by verifying the presence of potential interfering ions in Baijiu, including Br^−^, CO_3_^2−^, F^−^, SO_4_^2−^, Cl^−^, and H_2_PO_4_^−^. As shown in Figure 6A, compared to cyanide, the current response of other ions was less than 5.8%. The electrochemical determination of cyanide was not significantly impacted by the coexistence of interfering ions, indicating that the sensor possesses good anti−interference capability for the determination of cyanide. The repeatability of the sensor was evaluated through the determination of 2 ng/mL CN^−^ using the same Ag−Ni/Co−MOF−TCPP−Fc sensor. Good repeatability was shown by the current response from six repeated measurements, with only a 4.5% loss (Figure 6B).

### 3.6. Evaluation of CN^−^ in Fermented Grains and Baijiu

To verify the applicability of the Ag−Ni/Co−MOF−TCPP−Fc sensor, the designed sensor was applied to the determination and analysis of cyanide in fermented grains, Baijiu, river water, and mineral water. Baijiu samples were diluted to an alcohol content of below 20%, followed by the addition of different concentrations of cyanide standards (µg/mL). As can be seen from Table 2 and Table 3, the detected values were close to the added standard values, with recovery rates ranging between 98.5% and 103.3%. Besides, the relative standard deviations were low, ranging from 2.5% to 5.9%. The t and F statistical tests showed that there was no significant difference between the prepared electrochemical ratio sensor and GC−MS for the same sample. The electrochemical sensor is appropriate for cyanide assay in natural samples.

## 4. Conclusions

In summary, a simple and highly sensitive electrochemical sensor was developed based on the synthesis of a novel Ag−Ni/Co−MOF−TCPP−Fc for the determination of cyanides in Baijiu. Moreover, cyanide could be detected by the designed sensor within 4 min. The prepared Ag−Ni/Co−MOF−TCPP−Fc sensor exhibited an integrated reference signal, effectively establishing a ratio strategy that enhanced the stability of the sensor. The electron transport efficiency of Ag−Ni/Co−MOF was significantly improved by the ligands Fc−COOH and TCPP. It was indicated by experimental results that LOD was sufficient for monitoring cyanide levels in Baijiu, providing a new opportunity for the determination of cyanides in Baijiu.

## Figures and Tables

**Table 1:** Summary of this method and previous related studies.

Method	Response Time	Sensitivity	Determination Limit	Linear Range	Reference
Ion chromatography	−	0.0907	1 μg/L	10~160 μg/L	[5]
HPLC−MS	−	0.0053	0.07 μg/L	0.2~8.42 μg/L	[7]
Photoelectrochemical amperometry	−	0.0518	0.1 μM	0.1~60 μM	[42]
Fluorescence	4.8 min	2.45	2.41 μM	0~30 μM	[43]
Fluorescence	−	−	0.5 nM	1~50 nM	[44]
Fluorescence	−	−	0.11 μM	0.3~40 μM	[45]
Electrochemical	4 min	63.10612	0.052 ng/mL	0.196~44 ng/mL	This method

**Table 2:** Analyses of CN^−^−contaminated samples (mean ± SD).

Species		Method Comparison (µg/mL)		
		This Method	GC−MS	RSD
Fermented grains	Sample 1	0.021 ± 0.005 ^a^	0.020 ± 0.007 ^a^	5.6%
	Sample 2	0.020 ± 0.003 ^a^	0.021 ± 0.005 ^a^	5.9%
	Sample 3	0.018 ± 0.005 ^a^	0.017 ± 0.006 ^a^	4.7%
Baijiu	Sample 1	0.024 ± 0.007 ^a^	0.025 ± 0.006 ^a^	3.4%
	Sample 2	0.029 ± 0.003 ^a^	0.030 ± 0.004 ^a^	3.8%
	Sample 3	0.021 ± 0.004 ^a^	0.020 ± 0.002 ^a^	4.2%
River water	Sample 1	0.019 ± 0.003 ^a^	0.020 ± 0.002 ^a^	5.3%
	Sample 2	0.020 ± 0.002 ^a^	0.021 ± 0.002 ^a^	3.3%
	Sample 3	0.021 ± 0.003 ^a^	0.020 ± 0.004 ^a^	2.5%
Mineral water	Sample 1	Not detected	Not detected	−
	Sample 2	Not detected	Not detected	−
	Sample 3	Not detected	Not detected	−

Data are expressed as means ± SD of duplicate assays, Values followed different superscripts in the same column present significantly different (*p* < 0.05).

**Table 3:** Determination of CN^−^ using the proposed sensor.

Samples	Detected (µg/mL)	Added (µg/mL)	Found	Recovery
Fermented grains	0.021 ± 0.005	0.1	0.119 ± 0.005	98.9%
Baijiu	0.024 ± 0.007		0.128 ± 0.009	103.3%
River water	0.019 ± 0.006		0.121 ± 0.004	101.1%
Mineral water	Not detected		0.098 ± 0.003	98.5%

## Data Availability

Data are contained within the article.
